# The association of nucleotide‐binding oligomerization domain 2 gene polymorphisms with the risk of asthma in the Chinese Han population

**DOI:** 10.1002/mgg3.675

**Published:** 2019-04-04

**Authors:** Xulong Cai, Qiaolan Xu, Chenrong Zhou, Li Zhou, Weihua Dai, Guanchi Ji

**Affiliations:** ^1^ Department of Pediatrics Yancheng Third People's Hospital Yancheng China

**Keywords:** asthma, gene polymorphisms, IFN‐β, *NOD2*

## Abstract

**Background:**

Genetic background is one of the important risk factors for development of asthma. The nucleotide‐binding oligomerization domain 2 (*NOD2*) has been involved in the pathogenesis of asthma. The purpose of this study was to explore the relationship between *NOD2* gene polymorphisms and asthma susceptibility in the Chinese Han population.

**Methods:**

Children with asthma (*n* = 309) and Healthy children (*n* = 163) were recruited from Yancheng Third People's Hospital, Yancheng, China, between January 2016 and December 2017. The *NOD2* gene polymorphisms were measured by the Snapshot SNP genotyping assays. Genotyping was performed for 4 tag SNPs of *NOD2*. Serum IFN‐β levels were measured by ELISA.

**Results:**

The serum IFN‐β levels were significantly lower in Asthmatic children than those in the controls (*p* < 0.001). Low levels of IFN‐β may be related to the susceptibility to severe asthma. The rs3135499 C allele was associated with a significantly increased risk of asthma as compared with the rs3135499 A allele.

**Conclusion:**

The rs3135499 polymorphism of *NOD2* gene and IFN‐β may play a role in the pathogenesis of asthma.

## 1 INTRODUCTION

Asthma is a common chronic pulmonary disease. The clinical features are recurrent wheezing, shortness of breath, chest tightness, cough, and variable expiratory airflow limitation. Symptoms are often aggravated at night and in the morning. In 2016, 339 million people worldwide were suffering from asthma (Collaborators, 2017). Asthma is caused by complex environmental and genetic interactions (Ober & Vercelli, 2011). Exposure to allergens, tobacco smoke, air pollution, occupational risk factors, viral and bacterial infections, obesity, hygiene, stress, and toxic exposures may be a trigger for asthma (Toskala & Kennedy, 2015). Studies on twins suggest that genetic factors involve asthma (Koppelman, Los, & Postma, 1999; Laitinen, Rasanen, Kaprio, Koskenvuo, & Laitinen, 1998). Genome‐wide association studies of asthma have confirmed that the locus polymorphism of over 500 genes was involved in the pathogenesis of asthma (Macarthur et al., 2017).

Various T cell subtypes (Th1, Th2, Th9, Th17, NK, ILC2, and T regulatory cells) involved in asthma pathogenesis (Holgate et al., 2015). Nucleotide‐binding oligomerization domain‐containing protein 2 is a protein that is encoded by the *NOD2* (OMIM 605956) gene located on chromosome 16 in humans and spans a 39 kb genomic region comprised of 17 exons. *NOD2*‐deficient mice increased Toll‐like receptor 2–mediated T helper type 1 responses (Watanabe, Kitani, Murray, & Strober, 2004). Nucleotide binding and oligomerization domain 2 is an intracellular protein that recognizes bacterial muramyl dipeptide (Tigno‐Aranjuez & Abbott, 2012). This bacterial sensor NOD2 can trigger a strong antigen specific immune response with a Th2‐type polarization profile (Magalhaes et al., 2008). Moreover, NOD2 as a viral pattern recognition receptor that can sense viral to activate IFN‐β production and antiviral defense (Sabbah et al., 2009). The expression of interferon‐β in bronchial epithelial cells of asthma is impaired to infection with rhinovirus (Wark et al., 2005). NOD2 plays an important role in inflammatory and immune responses (Carneiro, Magalhaes, Tattoli, Philpott, & Travassos, 2008). *NOD2* has been involved in the development of Crohn's disease, early onset sarcoidosis, Blau syndrome, autoimmune disease, allergy, and asthma (Ni, Chen, Wu, Zhu, & Song, 2017).

Previous studies have found that *NOD2* polymorphism is associated with asthma in the German population (Kabesch et al., 2003; Weidinger et al., 2005). However, until now, the current studies failed to provide a basis for the genetic correlation of *NOD2* variations and asthma in the Chinese populations. Therefore, the aim of this research was to identify the role of *NOD2* polymorphisms in the genetic basis of asthma in the Chinese population and to evaluate the relationship between the *NOD2* polymorphisms and the serum level of interferon‐β.

## 2 MATERIALS AND METHODS

### 2.1 Ethical compliance

This research was conducted in accordance with the ethical standards of the Declaration of Helsinki. The research has been approved by the Ethics Committee of the Yancheng Third People's Hospital. Informed written consent was obtained from all parents.

### 2.2 Study subjects

The case‐control study included 163 controls and 309 asthmatic children. The asthmatic children in this study were in the clinical remission stage. Children were diagnosed for asthma according to the following criteria: cough, wheezing, shortness of breath, chest tightness, and lung function test. Controls were children without a history of allergy and family history of asthma. The controls underwent a routine medical checkup in the Medical Examination Center, Yancheng Third People's Hospital, Yancheng, China, between January 2016 and December 2017. All study subjects were of the Chinese Han population and resided in Yancheng, China.

### 2.3 DNA extraction and genotyping

Genomic DNA was extracted using the TIANamp Blood DNA Kit (Tiangen BiotechCo., Ltd., Beijing, China) following the manufacturers’ instructions and then stored at −80°C. SNP in the human *NOD2* (GenBank: AF178930.1) genes with minor allele frequencies >10% were selected from the HapMap Chinese data set. Tag SNPs were then selected by a tagger, using Haploview 4.2 software. The designs of PCR primers were carried out by online primer 3.0 software (http://primer3.ut.ee/). The SNaPshot was used to analyze genotypes of SNPs.

### 2.4 Serum IFN‐β determination

The quantity determination of serum IFN‐β levels was performed by IFN‐β Human ELISA Kit (Invitrogen) following the manufacturer's instructions.

### 2.5 Statistical analysis

For comparison of values between cases and controls, Student's *t* tests and the *χ* ^2^‐test were used. The Hardy–Weinberg equilibrium was tested for using *χ* ^2^‐test goodness of fit. Odds ratios (ORs) and 95% confidence intervals (CIs) were used for assessing the allele on the risk of asthma. The SPSS 17.0 was used for statistical analyses, and statistical significance was assumed at the *p* < 0.05 level. The statistical power to detect association of the polymorphisms with *NOD2* was 0.80 and was estimated with PASS 11 software (https://www.ncss.com).

## 3 RESULTS

### 3.1 Clinical characteristics of the study participants

There were no significant differences in the age and gender between patients and controls (*p* > 0.05) (Table 1). Asthma patients showed a significantly high rate of household smoking and recurrent respiratory infection (*p* < 0.05). Total IgE concentration in serum of the children with asthma was significantly higher than the controls (*p* < 0.05). Compared with the control group, the serum IFN‐β levels were significantly lower in the group of patients with asthma [(50.2 ± 15.6 pg/ml, *n* = 309) vs. (70.2 ± 14.7 pg/ml, *n* = 163); *t* = 13.483, *p* = 0.000].

**Table 1:** Clinical characteristics of the participants

Variable	Asthma patients	Control subjects	*p*
	*n* = 309	*n* = 163	
Age (mean ± *SD*)	10.7 ± 2.1	11.2 ± 2.6	0.409
Gender (M/F)	187/122	94/69	0.549
sIgE (IU/ml)	302.8 ± 87.5	61.3 ± 38.2	0.000
Household smoking	91	30	0.009
Recurrent respiratory infection	106	22	0.000
Atopy	216	0	–
Severity	45	0	–
Rhinitis	147	0	–
Medication	254	0	–
FEV_1_/FVC (%)	78.2 ± 6.4	–	–

### 3.2 The genotype and allele frequencies of NOD2 gene polymorphisms

Thirty‐four SNPs of *NOD2*, with minor allele frequencies >10%, were identified in the HapMap Chinese data set (Table 2), and all were captured by 4 tag SNPs of *NOD2*, using a tagger in Haploview software. For *NOD2*, pairwise tagging was performed at *r* ^2^ > 0.8, and the mean *r* ^2^ was 0.974. Next, genotyping was performed using the 4 tag SNPs. In the cases and the controls, the genotype distributions of rs1077861, rs3135499, rs1861759, and rs2111234 were consistent with the Hardy–Weinberg equilibrium (all *p* > 0.05).

**Table 2:** Tag and captured SNPs in the NOD2 gene

Tag SNPs	rs1077861	rs3135499	rs1861759	rs2111234
Captured SNPs	rs11642646	rs13332952	rs113656815	rs2111235
	rs17312836	rs9925315	rs79877183	
	rs11642482	rs3135500	rs1861757	
	rs11647841	rs8057341	rs61199363	
	rs8045009	rs4785449	rs79984321	
	rs34133110	rs4785225		
	rs10521209	rs7187857		
	rs748855	rs751271		
	rs8061960	rs9921146		
	rs7203691	rs6500328		
	rs2357791	rs8057341		
	rs1861758			
	rs4990643			

The distribution of genotypes and alleles frequencies of the 4 tag SNPs in the group of cases and the group of controls are shown in Table 3. Under codominant and dominant models, the genotype frequencies of the *NOD2* rs3135499 polymorphisms were statistically significant between the patients and the controls (*p* < 0.05). The rs3135499 C allele was associated with a significantly increased risk of asthma as compared with the rs3135499 A allele (OR = 1.762, 95% CI, 1.220–2.545, *p* = 0.002). However, the rs1077861, rs1861759, and rs2111234 SNPs were not significantly associated with asthma pathogenesis (*p* > 0.05).

**Table 3:** The distribution of genotype frequencies of NOD2 polymorphisms in asthma children and controls

SNPs	Model		Asthma	Control	OR (95%CI)	*p*
rs1077861	Codominant	TT	198	115	1	0.297
		AT	102	43	1.378 (0.902–2.105)	
		AA	9	5	1.045 (0.342–3.195)	
	Dominant	TT	198	115	1	0.157
		AT+AA	111	48	1.343 (0.892–2.022)	
	Recessive	AA	9	5	1	0.925
		TT+AT	300	158	1.055 (0.348–3.201)	
	Allele	T	498	273	1	0.233
		A	120	53	0.806 (0.565–1.149)	
rs3135499	Codominant	AA	183	122	1	0.003
		AC	116	37	2.090 (1.353–3.230)	
		CC	10	4	1.667 (0.511–5.435)	
	Dominant	AA	183	122	1	0.001
		AC+CC	126	41	2.049 (1.346–3.119)	
	Recessive	CC	10	4	1	0.634
		AA+AC	299	159	0.752 (0.232–2.437)	
	Allele	A	482	281	1	0.002
		C	136	45	1.762 (1.220–2.545)	
rs1861759	Codominant	AA	209	112	1	0.840
		AC	85	45	1.012 (0.660–1.553)	
		CC	15	6	1.340 (0.506–3.549)	
	Dominant	AA	209	112	1	0.812
		AC+CC	100	51	1.051 (0.699–1.580)	
	Recessive	CC	15	6	1	0.557
		AA+AC	294	157	0.749 (0.285–1.969)	
	Allele	A	503	269	1	0.671
		C	115	57	0.927 (0.653–1.316)	
rs2111234	Codominant	CC	129	77	1	0.247
		CT	146	75	1.162 (0.782–1.727)	
		TT	34	11	1.845 (0.884–3.852)	
	Dominant	CC	129	77	1	0.253
		CT+TT	180	86	1.249 (0.853–1.830)	
	Recessive	TT	34	11	1	0.135
		CC+CT	275	152	0.585 (0.288–1.188)	
	Allele	C	404	229	1	0.130
		T	214	97	0.800 (0.599–1.068)	

### 3.3 Distribution of IFN‐β between cases and controls

*NOD2* gene polymorphisms and clinical parameters had been further investigated for the impact of serum IFN‐β levels (Tables 4 and 5). We further found that severe asthma patients had lower levels of IFN‐β than nonsevere asthma. But, we failed to find any association of the rs1077861, rs3135499, rs1861759, and rs2111234 with serum level of IFN‐β.

**Table 4:** Distribution of IFN‐β between cases and controls

Model		Asthma		Control	
		IFN‐β levels (pg/ml)	*p*	IFN‐β levels (pg/ml)	*p*
Codominant	AA	50.7 ± 16.6	0.734	71.1 ± 14.2	0.334
	AC	49.6 ± 12.8		68.0 ± 16.4	
	CC	47.8 ± 25.5		63.0 ± 10.8	
Dominant	AA	50.7 ± 16.6	0.463	71.1 ± 14.2	0.182
	AC+CC	49.4 ± 14.1		67.5 ± 15.9	
Recessive	CC	47.8 ± 25.5	0.772	63.0 ± 10.8	0.327
	AA+AC	50.3 ± 15.2		70.4 ± 14.8	

**Table 5:** The distribution of NOD2 genotype and IFN‐β protein in different clinical characteristics

Group	Variable	Genotype			*p*	IFN‐β levels (pg/ml)	*p*
		AA	AC	CC			
Asthma							
	Household smoking						
	Positive	57	31	3	0.715	50.7 ± 16.0	0.687
	Negative	126	85	7		50.0 ± 15.5	
	Recurrent respiratory infection						
	Positive	65	37	4	0.755	50.6 ± 15.3	0.736
	Negative	118	79	6		50.0 ± 15.8	
	Atopy						
	Positive	122	86	8	0.304	50.8 ± 15.8	0.31
	Negative	61	30	2		48.8 ± 15.3	
	Severity						
	Positive	25	18	2	0.802	39.5 ± 16.1	0.000
	Negative	158	98	8		52.0 ± 14.8	
	Rhinitis						
	Positive	90	53	4	0.747	50.2 ± 15.3	0.979
	Negative	93	63	6		50.2 ± 16.0	
	Medication						
	Positive	152	93	9	0.659	50.0 ± 16.1	0.698
	Negative	31	23	1		50.9 ± 13.4	
Control							
	Household smoking						
	Positive	21	8	1	0.784	70.3 ± 16.9	0.964
	Negative	101	29	3		70.1 ± 14.2	
	Recurrent respiratory infection						
	Positive	16	6	0	0.646	70.1 ± 16.7	0.986
	Negative	106	31	4		70.2 ± 14.4	

## 4 DISCUSSION

Asthma is a common chronic respiratory disease in the world. Environmental and genetic factors affect the development of asthma. Environmental exposure to tobacco smoke is the most important risk factor for asthma, and causes airway inflammation (Sheikh, Pitts, Ryan‐Wenger, Mccoy, & Hayes, 2016). As a potential innate immune mechanism, the nucleotide‐binding oligomerization domain‐like receptors (NLRs) based inflammasome can increase the response to pollutants (Bauer, Diaz‐Sanchez, & Jaspers, 2012). As intracellular sensors, NLRs include 22 members in humans and 34 members in mice (Motta, Soares, Sun, & Philpott, 2015). NOD2 belongs to the NLR family and functions as a general sensor for both Gram‐positive and Gram‐negative bacteria by identifying muramyl dipeptide (Kufer, Banks, & Philpott, 2006). It was found that the physiological role of *NOD2* in antiviral defense was the enhanced respiratory syncytial virus pathogenesis, lung disease, and greater viral susceptibility through the study of *NOD2*‐deficient mice (Sabbah et al., 2009). NOD2 participates in host responses to infectious pathogens, including bacteria, viruses, and parasites (Al Nabhani, Dietrich, Hugot, & Barreau, 2017).

Genetic polymorphisms may be related to the development of diseases (Huang, 2015). There are some SNPs of *NOD2* that have been identified as susceptibility loci of Crohn's disease, including 1007 fs, G908R, P268S, and R702W (Cao et al., 2018). A research reported that the *NOD2* gene rs2066842 and rs2066843 polymorphisms showed a significant association with ulcerative colitis, but not with Crohn's in Indian patients (Pugazhendhi, Santhanam, Venkataraman, Creveaux, & Ramakrishna, 2013). Ahangari, Salehi, Salehi, & Khanahmad (2014) showed that the rs3135500 AA genotype had a significant association with risk of Colorectal cancer in the Iran population. The research data of Cao et al. suggest that the rs3135500 variant might increase the risk for multiple system atrophy. A previous study found that the rs751271 polymorphism was associated with inflammatory reactions in leprosy (Sales‐Marques et al., 2017). Weidinger et al., (2005) study found that the rs1077861 T allele decreased the risk of asthma, whereas the rs3135500 A allele was significantly associated with an increased risk of asthma.

Nod1 (Nucleotide‐binding oligomerization domain‐containing protein 1, encoded by the *NOD1* gene) and *NOD2* are important recognition receptors involved in inflammation and immune response(Elia, Tolentino, Bernardazzi, & de Souza, 2015). NOD1 and NOD2 conferred a upregulation of NF‐κB transactivation in transfected cells(Rosenstiel et al., 2006). *NOD1* insertion/deletion polymorphism was correlated with and inflammatory bowel disease in Caucasian populations(Lu, 2010). Previous research reported that *NOD1 *+* *32656 polymorphism is associated with elevated serum IgE levels(Hysi et al., 2005). The *NOD1 *+* *32656 locus insertion allele exhibit a significantly elevated production of IL‐1β and IL‐6(Plantinga et al., 2013). Three locus polymorphisms within the coding region of *NOD2*, G908R, R702W, and L1007fsinsC display a deficit in NF‐kB activation in response to bacterial components(Bonen et al., 2003; Rosenstiel et al., 2006). R702W, G908R, and Leu1007fsinsC polymorphisms in the *NOD2* gene were reported to be associated with sepsis susceptibility(Tekin et al., 2012). A study found that the *NOD2* rs3135499 polymorphism is associated with enhanced production of IL‐17A in human toxoplasmosis(Dutra et al., 2012). Therefore, it might be possible that mutations in *NOD1* or *NOD2* gene influence directly or indirectly to change in levels of inflammatory factors that may lead to an abnormal immune response.

In this study, we have analyzed the potential associations of polymorphisms in the *NOD2* gene with asthma in Chinese population. Among 4 tag SNPs of *NOD2* that were identified using tagger in Haploview software. The rs3135499 polymorphisms in the *NOD2* gene was significantly associated with asthma in the Chinese Han population. Furthermore, the rs3135499 C allele increased the risk of asthma as compared with the rs3135499 A allele. In addition, previous studies had found that rs3135499 polymorphisms involved in retinochoroiditis and leprosy(Dutra et al., 2012; Xiong et al., 2016). And the serum level of IFN‐β was significantly reduced in the cases as compared with the controls in this study. However, the distribution of the serum IFN‐β levels of individuals with AA, AC, and CC genotypes were no differences in the asthma group or the controls. These results suggested that rs3135499 polymorphisms may not affect the expression of serum IFN‐β. Previous studies had found IFN‐β expression was deficient in asthmatic patients (Sykes et al., 2012; Uller et al., 2010). A statistical significance was observed in the distribution of IFN‐β levels between severe asthma and nonsevere asthma patients. The result indicated that low levels of IFN‐β may be contribute to the susceptibility to severe asthma.

In summary, this study provided evidence that the *NOD2* gene rs3135499 polymorphism genotypes differed between children with asthma and healthy children in the Chinese Han population. The rs3135499 C allele as a risk factor may influence the development of asthma. Nonetheless, due to the limited sample size and the specific genetic characteristics of the Chinese population, the pathogenesis of *NOD2* in asthma needs further study to verify our results.

## CONFLICT OF INTEREST

All authors report no conflict of interest relevant to this article.
